# Supplementary material for: Bond-selective intensity diffraction tomography
Source: Nat Commun. 2022 Dec 15;13:7767. doi: 10.1038/s41467-022-35329-8 (PMC9755124; doi:10.1038/s41467-022-35329-8)
Supplement: Supplementary file 1 — Supplementary Information [file 41467_2022_35329_MOESM1_ESM.pdf]

Supplementary information for

Bond-Selective Intensity Diffraction Tomography

Jian Zhao[1,†,£], Alex Matlock[1,†,§], Hongbo Zhu[2,*], Ziqi Song[3], Jiabei Zhu[1], Biao Wang[2], Fukai Chen[4], Yuewei Zhan[5], Zhicong Chen[1], Yihong Xu[6], Xingchen Lin[2], Lei Tian[1,5,*] and Ji-Xin Cheng[1,5,6,*]

[1]Department of Electrical and Computer Engineering, Boston University, Boston, MA 02215, USA
[2]State Key Laboratory of Luminescence and Applications, Changchun Institute of Optics, Fine Mechanics and Physics, Chinese Academy of Sciences, Changchun 130033, China
[3]Aerospace Information Research Institute, Chinese Academy of Sciences, Beijing 100190, China
[4]Department of Biology, Boston University, Boston, MA 02215, USA
[5]Department of Biomedical Engineering, Boston University, Boston, MA 02215, USA
[6]Department of Physics, Boston University, Boston, MA 02215, USA
[†]These authors contribute equally to this work.
[£]Current affiliation: The Picower Institute for Learning and Memory, Massachusetts Institute of Technology, Cambridge, Massachusetts 02142, USA
[§]Current affiliation: Department of Mechanical Engineering, Massachusetts Institute of Technology, Cambridge, Massachusetts 02142, USA
*Corresponding authors.
*E-mail: jxcheng@bu.edu (J.X.C.); leitian@bu.edu (L.T.); zhbciomp@163.com (H.B.Z.)

BS-DPC imaging model

Here, we briefly summarize the DPC imaging theory and its application to the BS-IDT data. Details on DPC imaging method can be found in the literature[1]. Under the DPC imaging model, we consider the 2D measured intensity to be composed of the product of the object's complex transmittance $O(x, y)$ and incident field function $u_i(x, y|\mathbf{v_i})$ convolved with the objective's pupil function $P(\mathbf{v})$

$$I(x, y) = \iint |\mathcal{F}^{-1}\{\mathcal{F}\{u_i(x, y|\mathbf{v_i})O(x, y)\}P(\mathbf{v})\} |^2 d^2\mathbf{v_i}, \tag{1}$$

integrated over the discrete size of the illumination source occupying lateral spatial frequencies $\mathbf{v_i}$. This term accounts for the field contribution from each point in the illumination source interacting with the object as it propagates to the imaging plane. In DPC imaging, the illumination source is usually of low coherence occupying a half circle or annular circle function in the Fourier or far-field regime[1]. Under the Weak Object Approximation (WOA), DPC imaging assumes the object's complex transmission function can be linearized via a Taylor series expansion as $O(x, y) \approx 1 - \mu(x, y) + j\phi(x, y)$ where $\mu(x, y)$ and $\phi(x, y)$ represent the object's 2D absorption and phase information, respectively. In a similar form to BS-IDT, this assumption enables a linearization of the scattering model. A forward model can be developed relating the object's phase and absorption to the intensity spectra's cross-interference terms

$$\hat{I}(\mathbf{v}) = A\delta(\mathbf{v}) + H_\mu(\mathbf{v})\hat{\mu}(\mathbf{v}) + H_\phi(\mathbf{v})\hat{\phi}(\mathbf{v}), \tag{2}$$

with *A* measuring the total energy passing through the imaging system, phase $H_\phi(\mathbf{v})$ and absorption $H_\mu(\mathbf{v})$ TFs capturing the pupil function behavior between the object spectra and its complex conjugate

$$H_\mu(\mathbf{v}) = -\left[\iint S(\mathbf{v_i})P^*(\mathbf{v_i})P(\mathbf{v} + \mathbf{v_i})d^2\mathbf{v_i} + \iint S(\mathbf{v_i})P(\mathbf{v_i})P^*(\mathbf{v} - \mathbf{v_i})d^2\mathbf{v_i}\right], \tag{3a}$$

$$H_\phi(\mathbf{v}) = j\left[\iint S(\mathbf{v_i})P^*(\mathbf{v_i})P(\mathbf{v} + \mathbf{v_i})d^2\mathbf{v_i} - \iint S(\mathbf{v_i})P(\mathbf{v_i})P^*(\mathbf{v} - \mathbf{v_i})d^2\mathbf{v_i}\right], \tag{3b}$$

where $S(\mathbf{v_i})$ denotes the geometric source distribution in the Fourier plane. Of critical importance to DPC imaging is the phase TF's asymmetry and the absorption TF's symmetry between their first and second terms. For intensity image pairs using asymmetric illuminations, such as two half-circle illuminations occupying opposing halves of the Fourier plane, the subtraction of the two images will remove the absorption information of the image and double the phase contrast. This creates the DPC image that allows for the recovery of the object's phase information

$$\hat{I}_{DPC}(\mathbf{v}) = \mathcal{F}\left\{\frac{I_+(v) - I_-(v)}{I_0}\right\} \propto 2H_{\phi}(\mathbf{v})\hat{\phi}(\mathbf{v}), \quad (4)$$

where '+' and '-' represent intensity images from opposing illuminations in opposite halves of the Fourier plane and I_0 is the background intensity obtained from an image average. For BS-DPC imaging, we generated four DPC images from the 16 diode-laser illuminations in the BS-IDT datasets. Half-annular ring illumination geometries along the horizontal and vertical axes of the Fourier plane were chosen for this process to provide maximum bandwidth coverage with minimal spatial frequency information loss. The intensity images from each laser illumination within these half annular rings were summed. The images with corresponding asymmetric illuminations were subtracted to generate DPC images. We subsequently generated the DPC phase TFs for each image and reconstructed the object's 2D phase information using Tikhonov regularization after inverting the DPC forward model.

Lipid quantification

For the cancer cell's invasiveness analysis, we prepared two test groups, the T24 cell (invasive type) sample, and the SW780 cell (non-invasive type) sample. Both test groups were cultured and processed under the same conditions. For each test group, we chose 30 different single cells arbitrarily in the FOV to perform measurements at a wavenumber of 1745 cm^{-1}. Lipid contents absorb mid-IR radiation at this wavenumber strongly while other chemical components have no or significantly weak absorption, resulting in high lipid chemical signals. In addition, condensed lipid droplet areas have significantly higher RI than the surrounding medium, which enables an initial non-chemical filtering process. Based on the above facts, we first extracted 3D areas containing lipid droplets from the cold 3D reconstruction by local adaptive threshold. Then, we identified and extracted the true lipid droplets by comparing the filtered areas with the chemical imaging results. Finally, we calculated the lipid droplet volume by converting the total voxels into true spatial dimensions. We repeated the procedure above for 30 different cells per test group and made the box plot in Figure 3n.

BS-IDT resolution characterization

We characterized the resolution of BS-IDT and demonstrated the results in Figure S1. We used the fixed bladder cancer T24 cells as the test bed. By performing depth-resolved chemical imaging at 1745 cm^{-1} mid-IR wavenumber, we picked up two lipid droplets (Figure S1 a_1, b_1) to plot the lateral and axial line profiles (Figure S1 a_2, b_2). The FWHM of the lateral line profile is ~349 nm, and the FWHM of the axial line profile is ~1.082 μm. Here, we applied an additional halo artifact removal step to the chemical imaging data based on the work by Kandel *et al.* [2]. This process relies on the assumption that the halo effect is predominantly encoded into slowly varying spatial frequencies in the recovered object spectrum. By applying Hilbert transforms to the gradient of the phase along different directions in the image, the slowly varying halo artifacts are removed from the image. After applying these transforms and obtaining a collection of filtered images of the object, the maximum value at each pixel across these images is obtained to produce a halo-free image. For our BS-IDT chemical imaging data, the object's RI at each slice differs from the

corresponding phase only by a constant value. The object bandwidth still contains halo artifacts in low spatial frequencies. These factors allow us to apply the Hilbert transform approach equivalently to our sample for artifact removal. We performed a slice-wise artifact removal through our 3D RI reconstruction instead of the 2D phase image condition used in the original work[2].

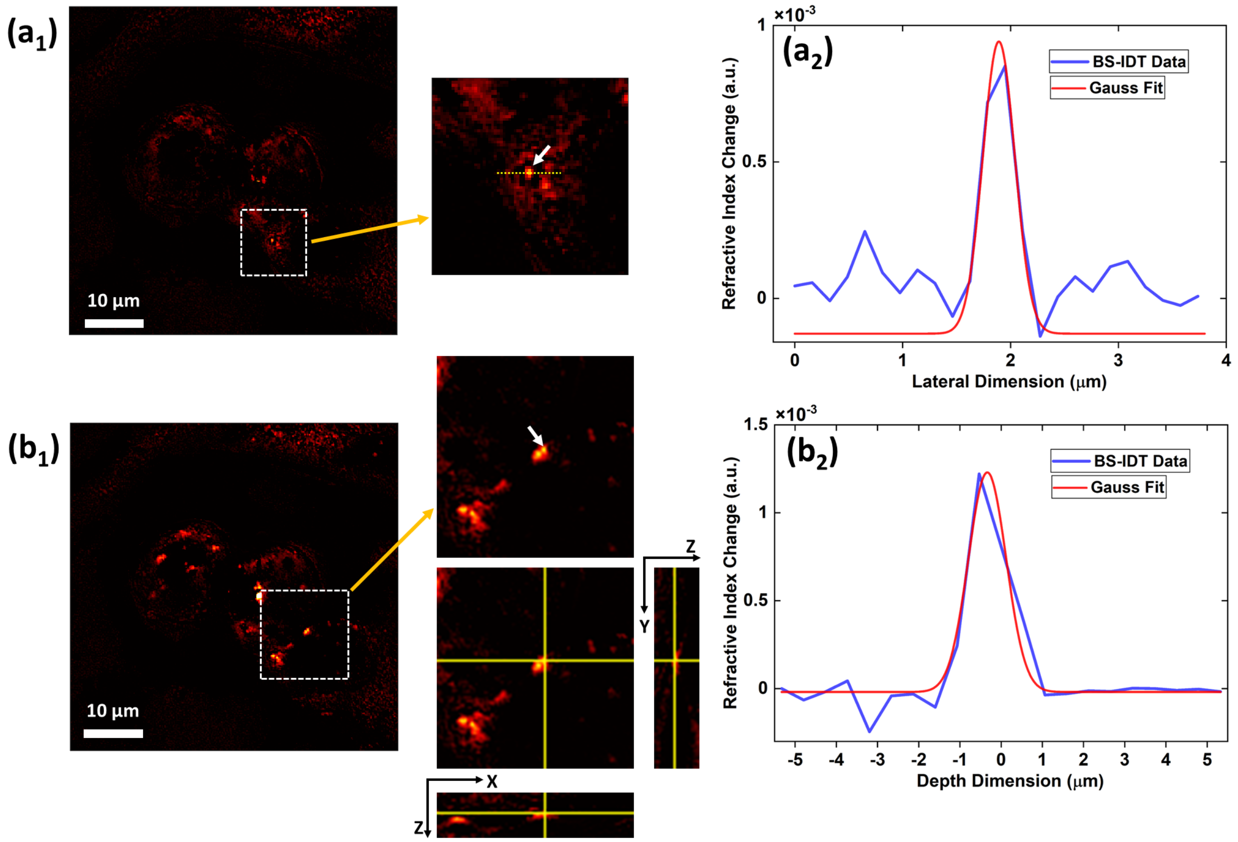

Figure S1. Resolution characterizations using lipid chemical imaging result from bladder cancer cells. The lipid chemical imaging data used here are further improved by halo artifacts removal processing. **(a_1)** The image corresponds to a depth of ~-1.065 µm. A selected lipid droplet is indicated by the white arrow shown in the inset. **(a_2)** Blue curve: extracted lateral profile along the yellow dashed line cross the selected lipid droplet in **(a_1)**; Red curve: Gaussian line shape fitting (FWHM: ~349 nm, R square coefficient=0.99) for the main peak corresponding to the selected lipid droplet. **(b_1)** The image corresponds to the depth of ~-0.532 µm. A selected lipid droplet is indicated by the white arrow shown in the inset. The orthogonal view of the selected lipid droplet is also demonstrated. “Z” indicates the depth direction. “X” and “Y” indicate lateral direction. **(b_2)** Blue curve: extracted depth profile from the selected lipid droplet’s peak signal in **(b_1)**; Red curve: Gaussian line shape fitting (FWHM:~1.082 µm, R square coefficient=0.95) for the main peak corresponding to the selected lipid droplet.

Heat dissipation measurement and simulation

We performed experimental and numerical simulations to estimate the exponential temporal decay time constants under different scenarios (Figure S2). The experimental measurement was performed using BS-IDT by characterizing the MIP-induced RI changes with respect to the pump-probe pulse time delay variations. The simulation was realized by COMSOL 5.4 (heat transfer in solid model). For all the simulation results shown in Figure S2, the bead is located in the center of a sphere that is composed of either D_2O or soybean oil. The sphere's diameter is 40 times larger than the bead’s diameter. The initial temperature of the sphere and the bead were set as 293 K and 298 K, respectively. The temperature of the sphere’s boundary was assumed to be constant. We simulated the temperature variation of the bead over time with different volumes

and chemical compositions. For experimental measurement, the sample is a polymethyl methacrylate (PMMA) bead with a volume of ~1.4 μm^3 immersed in D_2O. As a comparison, we simulated the thermal decay process for a PMMA bead of the same size. The experimental and simulation results are demonstrated in Figures S2 (a_1) and (a_2), respectively. A consistent thermal decay process is observed from both experimental and simulation results. We further investigated the thermal decay process for human fat beads of varying sizes immersed in two different types of media. We assumed that the volumes of the human fat beads vary from 1 μm^3 to 20 μm^3. As shown in Figure S2, the chemical compositions of the beads do not play a significant role in the decay time when the bead's volume is comparable to or smaller than 20 μm^3. Similar to the demonstration by Zong *et al.*[3], the larger size can significantly increase the temporal decay constant. In addition, beads immersed in heavy water demonstrate almost two times smaller temporal decay constant than those immersed in soybean oil.

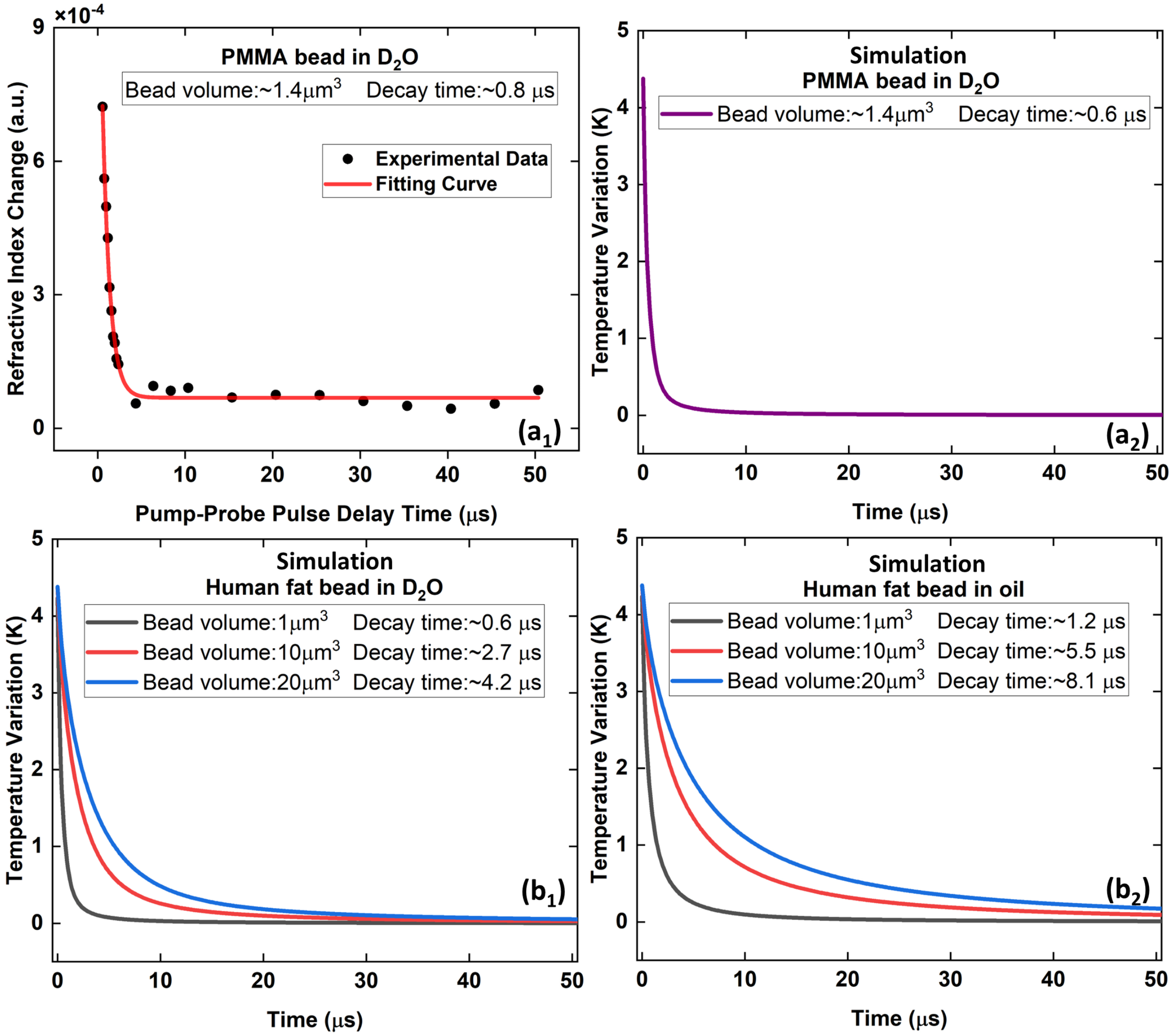

Figure S2. Experimental measurement and numerical simulations of thermal decay. (a_1) BS-IDT measurement result for a PMMA bead immersed in D_2O. **(a_2)** Simulation results for a PMMA bead with the same size as **(a_1)** immersed in D_2O. **(b_1)** Simulation results for human fat beads of different volumes immersed in D_2O. **(b_2)** Simulation results for human fat beads of different volumes immersed in soybean oil. The exponential temporal decay time constants are obtained by exponential decay curve fitting. All the R square coefficients for the fittings are equal to or larger than 0.97.

Supplementary References

1. Tian L, Waller L. Quantitative differential phase contrast imaging in an LED array microscope. *Opt Express* **23**, 11394-11403 (2015).

2. Kandel ME, Fanous M, Best-Popescu C, Popescu G. Real-time halo correction in phase contrast imaging. *Biomed Opt Express* **9**, 623-635 (2018).

3. Zong H, Yurdakul C, Bai Y, Zhang M, Ünlü MS, Cheng J-X. Background-Suppressed High-Throughput Mid-Infrared Photothermal Microscopy via Pupil Engineering. *ACS Photonics* **8**, 3323-3336 (2021).
