# Supplementary material for: Bond-selective intensity diffraction tomography
Source: Nat Commun. 2022 Dec 15;13:7767. doi: 10.1038/s41467-022-35329-8 (PMC9755124; doi:10.1038/s41467-022-35329-8)
Supplement: Supplementary file 3 — Description of Additional Supplementary Files [file 41467_2022_35329_MOESM3_ESM.pdf]

File name: Supplementary Movie 1

Description: 3D rendering of the fixed bladder cancer T24 cells under mitosis

File name: Supplementary Movie 2

Description: 3D rendering of the fixed C. *elegans* worm
