# Supplementary material for: Bond-selective intensity diffraction tomography
Source: Nat Commun. 2022 Dec 15;13:7767. doi: 10.1038/s41467-022-35329-8 (PMC9755124; doi:10.1038/s41467-022-35329-8)
Supplement: Supplementary file 6 — Reporting Summary [file 41467_2022_35329_MOESM6_ESM.pdf]

nature portfolio

Corresponding author(s): Ji-Xin Cheng

Last updated by author(s): Oct 20, 2022

Reporting Summary

Nature Portfolio wishes to improve the reproducibility of the work that we publish. This form provides structure for consistency and transparency in reporting. For further information on Nature Portfolio policies, see our Editorial Policies and the Editorial Policy Checklist.

Statistics

For all statistical analyses, confirm that the following items are present in the figure legend, table legend, main text, or Methods section.

n/a	Confirmed	
☐	☒	The exact sample size (*n*) for each experimental group/condition, given as a discrete number and unit of measurement
☐	☒	A statement on whether measurements were taken from distinct samples or whether the same sample was measured repeatedly
☒	☐	The statistical test(s) used AND whether they are one- or two-sided<br>*Only common tests should be described solely by name; describe more complex techniques in the Methods section.*
☒	☐	A description of all covariates tested
☒	☐	A description of any assumptions or corrections, such as tests of normality and adjustment for multiple comparisons
☐	☒	A full description of the statistical parameters including central tendency (e.g. means) or other basic estimates (e.g. regression coefficient) AND variation (e.g. standard deviation) or associated estimates of uncertainty (e.g. confidence intervals)
☒	☐	For null hypothesis testing, the test statistic (e.g. *F*, *t*, *r*) with confidence intervals, effect sizes, degrees of freedom and *P* value noted<br>*Give P values as exact values whenever suitable.*
☒	☐	For Bayesian analysis, information on the choice of priors and Markov chain Monte Carlo settings
☒	☐	For hierarchical and complex designs, identification of the appropriate level for tests and full reporting of outcomes
☒	☐	Estimates of effect sizes (e.g. Cohen's *d*, Pearson's *r*), indicating how they were calculated

Our web collection on statistics for biologists contains articles on many of the points above.

Software and code

Policy information about availability of computer code

Data collection	Data were collected by customized MATLAB code.
Data analysis	Data were processed by customized MATLAB code and Fiji ImageJ Version 1.53c. The code is available at https://github.com/buchenglab/BS-IDT.

For manuscripts utilizing custom algorithms or software that are central to the research but not yet described in published literature, software must be made available to editors and reviewers. We strongly encourage code deposition in a community repository (e.g. GitHub). See the Nature Portfolio guidelines for submitting code & software for further information.

Data

Policy information about availability of data

All manuscripts must include a data availability statement. This statement should provide the following information, where applicable:

- Accession codes, unique identifiers, or web links for publicly available datasets
- A description of any restrictions on data availability
- For clinical datasets or third party data, please ensure that the statement adheres to our policy

All the data related to the work is available upon reasonable request to the corresponding author. All bladder cancer cell reconstruction data and Caenorhabditis elegans reconstruction data related to displayed images in the manuscript are available at https://github.com/buchenglab/BS-IDT_Data. All the source data related to line plots and box plots are submitted to Nature Communications and are available.

Human research participants

Policy information about studies involving human research participants and Sex and Gender in Research.

Reporting on sex and gender	This research does not involve human research participants.
Population characteristics	not applicable
Recruitment	not applicable
Ethics oversight	not applicable

Note that full information on the approval of the study protocol must also be provided in the manuscript.

Field-specific reporting

Please select the one below that is the best fit for your research. If you are not sure, read the appropriate sections before making your selection.

☒ Life sciences ☐ Behavioural & social sciences ☐ Ecological, evolutionary & environmental sciences

For a reference copy of the document with all sections, see nature.com/documents/nr-reporting-summary-flat.pdf

Life sciences study design

All studies must disclose on these points even when the disclosure is negative.

Sample size	For mid-infrared spectroscopy fidelity, we need to use the same sample with a size of one since we need to measure the same sample by comparing our system with the ground truth system. The ground truth is from the golden standard system, the Fourier-transform infrared spectroscopy (FTIR). By comparing with FTIR measurements, we confirmed our mid-infrared spectroscopy fidelity, which is also standard procedure in the chemical imaging community. For biological sample mid-infrared spectra measurements, the goals are to extract the spectra from certain areas of the same sample and demonstrate the chemical contents based on the absorption signature of the mid-infrared spectra. Amide bands and lipid band are two important bands for biological systems, which are the reason that was highlighted in our manuscripts. Our chemical imaging results showed which areas in the same sample had rich protein (Amide bands) or lipid contents. Then, we can extract the mid-infrared spectra from them. This process just needs one sample, and no sample size calculation is needed. This is also widely accepted in the chemical imaging community. For chemical imaging of cancer cells and C. elegans, we need to compare the molecular-specific imaging contrast within the same sample, which is a standard method in the chemical imaging community. No sample size calculation is required for the chemical imaging of cells and C. elegans. For differentiating different types of cancer cells, box plot analysis requires a sample size of 5, while we picked up a modest large sample size of 30. This sample size of 30 is large enough for box plot analysis. These numbers guarantee statistical significance. We also chose these numbers by referring to certain reliable literatures, for example, the reference "Visualizing samples with box plots, Nature Methods, 11, 119-120, 2014, Martin Krzywinski and Naomi Altman".
Data exclusions	No data was excluded from analysis.
Replication	The data are available at https://github.com/buchenglab/BS-IDT_Data. The code is available at https://github.com/buchenglab/BS-IDT.
Randomization	Cells for imaging and other analysis purposes are randomly picked up within the field of view.
Blinding	The investigators were blinded to group allocation during data collection and analysis.

Reporting for specific materials, systems and methods

We require information from authors about some types of materials, experimental systems and methods used in many studies. Here, indicate whether each material, system or method listed is relevant to your study. If you are not sure if a list item applies to your research, read the appropriate section before selecting a response.

Materials & experimental systems

n/a	Involved in the study	
☒	☐	Antibodies
☐	☒	Eukaryotic cell lines
☒	☐	Palaeontology and archaeology
☐	☒	Animals and other organisms
☒	☐	Clinical data
☒	☐	Dual use research of concern

Methods

n/a	Involved in the study	
☒	☐	ChIP-seq
☒	☐	Flow cytometry
☒	☐	MRI-based neuroimaging

Eukaryotic cell lines

Policy information about cell lines and Sex and Gender in Research

Cell line source(s)	Authenticated T24 and SW780 cells were obtained from the American Type Culture Collection (ATCC).
Authentication	Authenticated T24 and SW780 cells were obtained from the ATCC. The cell lines have not been authenticated after purchase.
Mycoplasma contamination	Mycoplasma contamination test has not been tested after purchase.
Commonly misidentified lines (See ICLAC register)	None

Animals and other research organisms

Policy information about studies involving animals; ARRIVE guidelines recommended for reporting animal research, and Sex and Gender in Research

Laboratory animals	Caenorhabditis elegans (daf-2 (e1370) mutant strain) were were purchased directly from the Caenorhabditis Genetics Center at the University of Minnesota.
Wild animals	No wild animals were used in the study.
Reporting on sex	Sex is not considered in this research. Sex does not play a role in this research.
Field-collected samples	No field-collected samples were used in the study.
Ethics oversight	No ethical approval was needed for this study.

Note that full information on the approval of the study protocol must also be provided in the manuscript.
